# Gag-Vpu cross talk modulating HIV-1 envelope incorporation and infectivity in cell-type dependent manner

**DOI:** 10.1186/1471-2334-12-S1-P49

**Published:** 2012-05-04

**Authors:** Archana Gautam, Ajit Patil, Jayanta Bhattacharya

**Affiliations:** 1 Department of Molecular Virology, National AIDS Research Institute, G-73, MIDC, Bhosari, Pune-411026, India

## Introduction

HIV-1 Vpu plays important role in enhancing virus release and CD4 down-modulation. We investigated the effect of Vpu-Gag cross talk on envelope incorporation and assembly in different cell types including primary cells.

## Methods

We introduced Vpu start codon mutation and point substitution in p17 Gag matrix (L30E) in HIV-1 pNL-AD8 through PCR. Progeny viruses were produced in two cell-types: 293T and HeLa by transfection. Infectivity potential of Vpu+/Vpu- viruses carrying Gag (L30E) mutation was assessed in TZM-bl cell line and their replication potential in peripheral blood mononuclear cells (PBMC) and monocyte-derived macrophages. The effect of mutation on virus release, envelope incorporation and infectivity was determined by RT ELISA and Western blot.

## Results

The amount of virus release from 293T and HeLa cells was similar in Vpu+/Vpu- constructs carrying Gag (L30E) mutation but the infectivity potential of viruses varied, showing enhanced infectivity of Vpu(-) Gag L30E viruses produced from 293T cell and HeLa cells. Envelope incorporation assay using 293T cells revealed that Vpu+ viruses with L30E mutation showed inefficient incorporation of HIV-1 envelope on cell-free virions whereas viruses with Vpu-/ L30E mutation resulted in efficient envelope incorporation and thereby increasing their infectivity potential. While similar results were obtained with PBMC and macrophages as found with 293T and HeLa cells, these effects were found to vary in different cell types.

## Conclusion

HIV-1 envelope incorporation and infectivity is dependent on cross talk between p17 Gag, Vpu and Envelope and the effect of Gag p17 mutation on envelope is Vpu-mediated.

